# Development and Validation of Quantile Regression Forests for Prediction of Reference Quantiles in Handgrip and Chair‐Stand Test

**DOI:** 10.1002/jcsm.13868

**Published:** 2025-06-17

**Authors:** Giulia Giordano, Luca Mastrantoni, Francesco Landi, The Lookup 8+ Study Group

**Affiliations:** ^1^ Department of Geriatrics, Orthopedics and Rheumatological Sciences Fondazione Policlinico Universitario Agostino Gemelli, IRCCS Rome Italy; ^2^ Department of Geriatrics, Orthopedics and Rheumatological Sciences Università Cattolica del Sacro Cuore Rome Italy; ^3^ Medical Oncology Università Cattolica del Sacro Cuore Rome Italy

**Keywords:** chair‐stand test, EWGSOP2, handgrip strength, machine learning, sarcopenia

## Abstract

**Background:**

Muscle strength is one of the key components in the diagnosis of sarcopenia. The aim of this study was to train a machine learning model to predict reference values and percentiles for handgrip strength and chair‐stand test (CST), in a large cohort of community dwellers recruited in the Longevity check‐up (Lookup) 8+ project.

**Methods:**

The longevity checkup project is an ongoing initiative conducted in unconventional settings in Italy from 1 June 2015. Eligible participants were 18+ years and provided written informed consent. After a 70/20/10 split in training, validation and test set, a quantile regression forest (QRF) was trained. Performance metrics were *R*‐squared (*R* ^2^), mean squared error (MSE), root mean squared error (RMSE) and mean Winkler interval score (MWIS) with 90% prediction coverage (PC). Metrics 95% confidence intervals (CI) were calculated using a bootstrap approach. Variable contribution was analysed using SHapley Additive exPlanations (SHAP) values. Probable sarcopenia (PS) was defined according to the European Working Group on Sarcopenia in Older People 2 (EWGSOP2) criteria.

**Results:**

Between 1 June 2015 and 23 November 2024, a total of 21 171 individuals were enrolled, of which 19 995 were included in our analyses. In the overall population, 11 019 (55.1%) were females. Median age was 56 years (IQR 47.0–67.0). Five variables were included: age, sex, height, weight and BMI. After the train/validation/test split, 13 996 subjects were included in the train set, 4199 in validation set and 1800 in the test set. For handgrip strength, the *R* ^2^ was 0.65 (95% CI 0.63–0.67) in the validation set and 0.64 (95% CI 0.62–0.67) in the test set. PCs were 91.5% and 91.2%, respectively. For CST test, the *R* ^2^ was 0.23 (95% CI 0.20–0.25) in the validation set and 0.24 (95% CI 0.20–0.28) in the test set. The PCs were 89.5% and 89.3%. Gender was the most influential variable for handgrip and age for CST. In the validation set, 23% of subjects in the first quartile for handgrip and 13% of subjects in the fourth quartile for CST test met criteria of PS.

**Conclusions:**

We developed and validated a QRF model to predict subject‐specific quantiles for handgrip and CST. These models hold promise for integration into clinical practice, facilitating cost‐effective and time‐efficient early identification of individuals at elevated risk of sarcopenia. The predictive outputs of these models may serve as surrogate biomarkers of the aging process, capturing functional decline.

## 1 Introduction

The global population of older adults is rapidly increasing, as the need to study the mechanisms of aging and its associated conditions. Among these, sarcopenia [1] is of particular concern due to its association with adverse outcomes, including falls, fractures, physical disability and increased mortality [2]. As defined by the European Working Group on Sarcopenia in Older People 2 (EWGSOP2), sarcopenia has evolved significantly in its diagnostic criteria over the years. Initially characterized by low muscle mass alone, the 2019 EWGSOP consensus introduced a refined definition, emphasizing low muscle strength as the principal diagnostic criterion, complemented by assessments of muscle quantity and quality [3]. Specific cutoff values for grip strength and the chair stand test (CST) are pivotal to identifying sarcopenia in clinical practice [3, 4]: grip strength, measured using a dynamometer, provides a rapid and practical indicator of upper body strength and the CST evaluates lower body strength by recording the time required for an individual to rise five times from a seated position without arm assistance. Current reference values for these tests are derived from population‐level data, generally stratified by gender and age [5, 6]. In this scenario, a more personalized approach could be beneficial: These thresholds may fail to detect individuals with early reductions in strength when their values do not yet fall below the established cutoffs, especially in younger subjects.

To address these gaps, machine learning (ML) models offer a promising solution. By capturing complex, nonlinear relationships and interactions between predictive variables, ML models can overcome traditional limitations and enhance diagnostic precision, providing individualized reference values [7, 8]. However, in standard regression tasks, only point estimates are generally calculated, which do not provide any information regarding the dispersion of observations around the predicted value. In this scenario, quantile regression forests (QRF), a nonparametric, tree‐based ensemble method can directly estimate conditional quantiles, naturally providing uncertainty estimation [9].

The aim of this study was the development and validation of a QRF model capable to predict reference values and quartiles for handgrip and CST. By providing personalized predictions alongside reference percentiles, the proposed model seeks to facilitate the early identification of individuals at higher risk of sarcopenia, supporting timely interventions.

## 2 Methods

The Longevity check‐up 8+ project (Lookup 8+) is an ongoing initiative supported by the Department of Geriatrics of the Università Cattolica del Sacro Cuore and Fondazione Policlinico Universitario ‘Agostino Gemelli’ IRCCS (Rome, Italy). The study protocol received approval from the Ethical Committee of Università Cattolica del Sacro Cuore (Protocol No. A.1220/ce/2011); further details have been previously published [10]. The study is based on a retrospective analysis of a longitudinal cohort and describes the development of prediction models in accordance with the guidelines for transparent reporting of multivariable prediction models for individual prognosis or diagnosis—Artificial Intelligence (TRIPOD‐AI, Table S6) [11].

### 2.1 Inclusion Criteria

All participants were at minimum 18 years of age and provided written informed consent prior to enrolment. Exclusion criteria included self‐reported pregnancy, inability to perform physical performance tests, refusal to undergo capillary blood testing to measure total cholesterol and glycemia, and inability or unwillingness to provide written informed consent. The Lookup 8+ study participants recruitment was conducted in unconventional settings, including exhibitions, shopping centres, social gatherings and prevention campaigns organized by Università Cattolica del Sacro Cuore, aiming to include unselected participants and enhance generalizability of findings.

### 2.2 Measurement of Variables and Endpoints

A standard stadiometer and an analog scale were used to measure standing height and body weight. The body mass index (BMI) was determined as the ratio between body weight (kg) and the square of height (m^2^). Data on dietary and exercise habits and other lifestyle behaviours were collected using a structured interview. Handgrip strength and CST assessments were conducted according to EWGSOP2 recommendations [3]. Trained investigators performed all evaluations [12]. Handgrip strength assessment was performed with subject sitting on a chair, with a neutral position of the hand, elbow flexed at 90°, arm close to the body; maximum contraction value was assessed with a hydraulic dynamometer (North Coast Hand Dynamometer; North Coast medical Inc., Morgan Hill, CA, United States). Participants performed one familiarization trial before testing. Handgrip strength was measured in both hands, and the highest value was used for the analysis [13]. The CST test was also performed asking participants to stand up from a chair with their arms folded across the chest five consecutive times as quickly as possible. The chair height was chosen to minimize the influence of chair seat height on the test results [14]. The time taken to complete the five repetitions was measured using a handled stopwatch and used for the analysis [15]. Probable sarcopenia (PS) was considered according to EWGSOP2 criteria, defined by grip strength < 27 kg for men and < 16 kg for women or chair stand test execution time > 15 s for five rises [3].

### 2.3 Statistical Analysis and Sample Size

The characteristics of the study participants were summarized using descriptive statistics. Continuous variables were expressed as median values with interquartile ranges (IQR), and categorical variables were reported as absolute counts and percentages. Descriptive statistics were stratified by gender to present demographic and clinical characteristics. Differences between categorical variables were assessed using Chi Squared or Fisher's exact test, whereas differences between continuous variables were evaluated using the Mann–Whitney *U* test. Correlation between variables was assessed using the Spearman correlation coefficient (*ρ*). The Brown–Forsythe test was used to compare variances between groups. A Uniform Manifold Approximation and Projection (UMAP) analysis was performed to visually inspect patients' characteristics and preliminarily identify potential clusters based on clinical features. Although no formal sample size calculation was performed in advance, the large number of included patients and the models' performances suggest that an adequate number of observations per variable was reached. Statistical analyses were performed using R Studio (ver 4.2.2) and Python (ver 3.10). All hypotheses were two‐sided, and *p* < 0.05 was considered statistically significant, except when otherwise specified.

### 2.4 Preprocessing

Samples with missing data for at least one of the following variables were excluded: age, sex, height, weight, handgrip strength or CST performance, and a complete case approach was used for model training. The dataset was randomly split into training, validation and test sets using a 70/20/10 proportion. This partition was used to ensure a large enough training set for model development and hyperparameter tuning and a 20% validation set to assess model performance. Given the large sample size, a 10% hold‐out test set was considerate adequate to ensure robust statistical evaluation. For model development and clinical interpretation, the variables age, gender, height, weight and BMI were chosen based on potential clinical utility and broad real‐world availability. Age, weight, height and BMI were considered as numerical variables whereas gender and PS as binary variables. The two endpoints were treated as continuous numerical variables, configuring a supervised regression task. Categorical variables were encoded using one‐hot encoding, and continuous variables were scaled using *z*‐score standardization as a preprocessing step.

### 2.5 Model Development and Evaluation

A QRF model [9] was trained using the quantile‐forest package, including in the pipeline hyperparameter tuning via Bayesian optimization with a five fold‐cross validation (CV) approach [16]. Using optuna [17], the following hyperparameters were tuned: number of trees (n_estimators, range 100–1000), maximum depth of trees (max_depth, range 3–20) and minimum number of samples require to be at a leaf node (min_sample_leaf, range 20–100). Optimization was run for 30 trials using the Tree‐structured Parzen Estimator sampler. For comparison, a standard random forest (RF) model was also trained using the sklearn package. Hyperparameters optimization was performed using the *R*‐squared (*R* ^2^) as target metric, aiming to maximize the explained variance. For each model, we evaluated the *R* ^2^, the mean squared error (MSE), the root mean squared error (RMSE) and the mean Winkler interval score (MWIS) with 90% prediction coverage (PC) [18]. Five‐thousand bootstrap replicates were adopted to calculate 95% confidence intervals (CI) for the *R* ^2^, MSE and RMSE. Two‐thousand bootstrap replicates were used for 95% CI and prediction intervals (PI) stratified to gender. Percentiles were calculated using the quantile argument of the predict function. The model with the highest mean *R* ^2^ across the cross‐validation folds of the train set was selected as the best‐performing model and evaluated on validation and test set. Agreement between actual and predicted values was visually assessed using actual versus predicted plots, and the distribution of residuals was examined. The F1 score was used to choose the prediction threshold in the test set. The cutoff was evaluated in the validation and test set, comparing the F1 score, the confusion matrix and accuracy.

### 2.6 Variable Contribution

To favour model explainability and facilitate the clinical interpretation of the results, Shapley Additive Explanations (SHAP) values were calculated using the SHAP package [19]. Beeswarm plots were generated to visualize the distribution of SHAP values across all features, and the absolute mean contributions of the features were also inspected. Dependence plots were utilized to explore potential interactions between variables.

## 3 Results

### 3.1 Patients' Population

Between 1 June 2015 and 23 November 2024, a total of 21 171 individuals were enrolled, of which 19 995 had no missing values in the variables of interest and were included in our analysis. Subjects' characteristics are reported in Table 1. In the overall population, 11 019 (55.1%) were female and 8976 (44.9%) males. Median age was 56 years (IQR 47.0–67.0). Median overall grip strength value in males was 40.0 kg (IQR 34.0–46.7) and in females 24.0 kg (IQR 20.4–28.0). The median time for the CST was 7.4 s (IQR 6.2–8.9): Male's median value was 7.3 s (IQR 6.1–8.7) and female's 7.5 s (IQR 6.2–9.0). After the train/validation/test split, 13 996 subjects were included in the train set, 4199 in validation set and 1800 in the test set. Patients' characteristics were well balanced between the three cohorts (Table S1).

**TABLE 1:** Patients' characteristics.

	Overall (*n* = 19 995)	Female (*n* = 11 019)	Male (*n* = 8976)	Missing
Age (years)	56.0 [47.0–67.0]	56.0 [47.0–66.0]	57.0 [47.0–67.0]	0
Active smoke				65
No	16 200 (81.0)	8986 (81.6)	7214 (80.4)	
Yes	3730 (18.7)	1997 (18.1)	1733 (19.3)	
Physical activity				89
No	8126 (40.6)	4822 (43.8)	3304 (36.8)	
Yes	11 780 (58.9)	6137 (55.7)	5643 (62.9)	
Weight (kg)	70.0 [60.0–80.0]	62.0 [56.0–70.0]	78.0 [71.0–86.0]	0
Height (cm)	168.0 [160.0–175.0]	162.0 [157.0–167.0]	175.0 [170.0–180.0]	0
Body mass index (kg/m^2^)	24.6 [22.2–27.4]	23.6 [21.2–26.7]	25.5 [23.6–28.0]	0
Systolic blood pressure (mmHg)	122.0 [110.0–135.0]	120.0 [110.0–130.0]	130.0 [120.0–140.0]	617
Diastolic blood pressure (mmHg)	76.0 [70.0–80.0]	74.0 [70.0–80.0]	80.0 [70.0–85.0]	621
Cholesterol (mg/dL)	197.0 [174.0–222.0]	201.0 [180.0–225.0]	190.0 [166.0–218.0]	968
Glucose (mg/dL)	100.0 [91.0–113.0]	99.0 [91.0–111.0]	101.0 [91.0–114.0]	379
Calf circumference (cm)	36.0 [33.8–38.0]	35.0 [33.0–37.0]	37.0 [35.0–39.0]	0
Handgrip (kg)	29.5 [23.0–40.0]	24.0 [20.4–28.0]	40.0 [34.0–46.7]	0
Chair stand test (s)	7.4 [6.2–8.9]	7.5 [6.2–9.0]	7.3 [6.1–8.7]	0
Sarcopenia EWGSOP2				0
No	18 553 (92.8)	10 274 (93.2)	8279 (92.2)	
Yes	1442 (7.2)	745 (6.8)	697 (7.8)	

*Note:* Continuous values are represented as median [IQR]. Categorical variables as *n* (%).

Abbreviations: EWGSOP2, European Working Group on Sarcopenia in Older People2 criteria; IQR, interquartile range.

### 3.2 Train Set Exploratory Analysis

**FIGURE 1:**
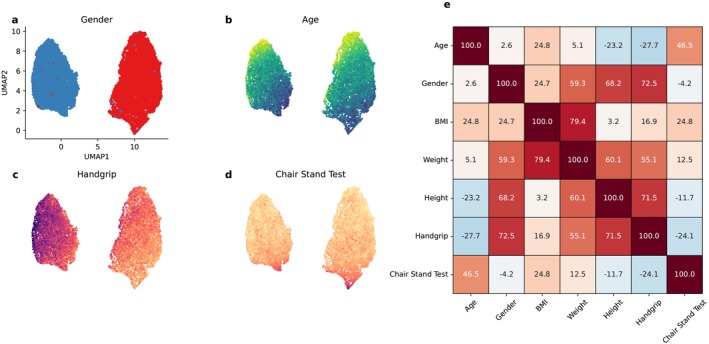
Train set exploratory analysis. (a–d) After preprocessing, UMAP (number of neighbours = 15) was applied to the train set, and points were coloured according to the different variables. For age, darker colours (blues) correspond to older age. For handgrip and chair stand test, darker colours (purple) correspond to higher values. (e) Correlation heatmap displaying Spearman correlation coefficients. Red shades indicate positive correlations, whereas blue shades represent negative correlations.

After UMAP, as expected, we observed two clear clusters of subjects corresponding to gender (Figure 1a). Within the low dimensional space, we observed a gradient along the UMAP axes both for age (with younger observations predominantly clustered in the upper region and older subjects concentrated towards the bottom; Figure 1b). A similar trend was observed for handgrip: stratified by gender, lower values were mapped in the upper right corner of the corresponding gender regions (Figure 1c). This showed a partial overlap with the CST values, mapped in the lower part of the embedding space (Figure 1d). As expected, we observed a negative correlation between handgrip and CST, and a minimal correlation between CST and gender (Figure 1e).

In the train set, we observed a significant difference in variances between females and male of the handgrip (35.51 vs. 90.07, *p* value 3.9 × 10^−242^) and the CST (6.31 vs. 4.78, *p* value 4.6 × 10^−9^). For handgrip, we observed a negative trend for variance values according to age bins, with a strong negative correlation both for males (Spearman rho, *ρ* = −0.96) and females (*ρ* = −0.89) (Figure S1). The opposite trend was observed for CST, with higher variances for older subjects (*ρ* = 0.96 for males and *ρ* = 0.90 for females) (Figure S2).

### 3.3 Handgrip Model

**FIGURE 2:**
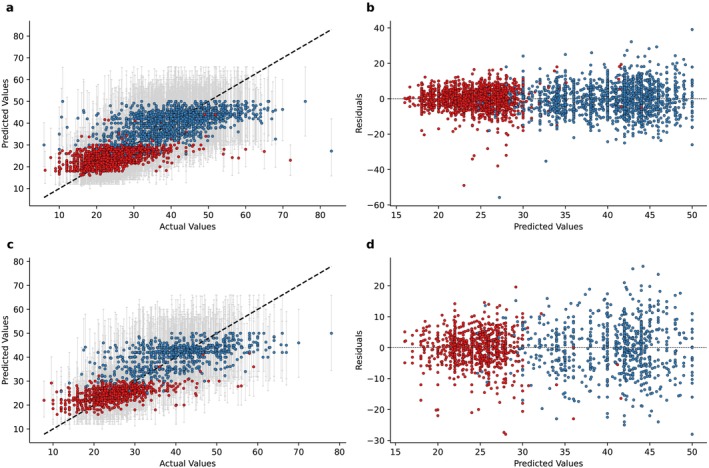
Model evaluation: handgrip. (a) Comparison between predicted values (*y* axis) and actual values (*x* axis) in the validation set. Error bars indicate the 5th and 95th percentile. (b) Comparison between residuals (*y* axis) and predicted values (*x* axis) in the validation set. (c) Comparison between predicted values (*y* axis) and actual values (*x* axis) in the test set. Error bars indicate the 5th and 95th percentile. (d) Comparison between residuals (*y* axis) and predicted values (*x* axis) in the test set.

For handgrip prediction, the QRF model achieved a mean cross‐validated *R* ^2^ of 0.65 in the training set, with a MSE of 42.54 and a RMSE of 6.52 (Table 2a and Figure S3). In the validation set, the *R* ^2^ was 0.65 (95% CI 0.63–0.67), with a MSE of 43.30 (95% CI 40.22–46.71) and a RMSE of 6.58 (95% CI 6.34–6.83). Consistent results were observed in the test set: The *R* ^2^ was 0.64 (95% CI 0.62–0.67), with a MSE of 45.34 (95% CI 41.59–49.22) and a RMSE of 6.58 (95% CI 6.45–7.02). The MWIS scores were 27.18 (PC: 91.5%) in the validation set and 28.05 (PC: 91.2%) in the test set, with similar values in males and females. The actual versus predicted values plots and residual plots are shown in Figures 2a–d and S4.

**TABLE 2:** Model performance of quantile random forest (QRF) regressor stratified by set and gender.

(a) Handgrip					
Set	*R* ^2^	MSE	RMSE	MWIS	PC
Train	0.65*	42.54*	6.52*	26.06	92.0%
Validation	0.65 (95% CI 0.63–0.67)	43.30 (95% CI 40.22–46.71)	6.58 (95% CI 6.34–6.83)	27.18	91.5%
Female	0.29 (95% CI 0.25–0.33)	27.73 (95% CI 24.46–31.45)	5.27 (95% CI 4.95–5.61)	22.34	91.1%
Male	0.28 (95% CI 0.24–0.32)	62.67 (95% CI 57.43–68.74)	7.92 (95% CI 7.58–8.29)	33.21	91.9%
Test	0.64 (95% CI 0.62–0.67)	45.34 (95% CI 41.59–49.22)	6.58 (95% CI 6.45–7.02)	28.05	91.2%
Female	0.28 (95% CI 0.22–0.33)	28.78 (95% CI 24.80–33.02)	5.36 (95% CI 4.98–5.75)	22.96	91.8%
Male	0.31 (95% CI 0.25–0.36)	64.56 (95% CI 58.20–71.48)	8.04 (95% CI 7.63–8.45)	33.97	90.4%

(b) Chair stand test					
Set	*R* ^2^	MSE	RMSE	MWIS	PC
Train	0.24*	4.27*	2.07*	7.72	92.8%
Validation	0.23 (95% CI 0.20–0.25)	4.68 (95% CI 4.12–5.32)	2.16 (95% CI 2.03–2.31)	8.70	89.5%
Female	0.23 (95% CI 0.19–0.26)	5.22 (95% CI 4.40–6.25)	2.28 (95% CI 2.10–2.50)	9.23	89.5%
Male	0.21 (95% CI 0.17–0.25)	4.02 (95% CI 3.41–4.67)	2.00 (95% CI 1.85–2.16)	8.04	89.6%
Test	0.24 (95% CI 0.20–0.28)	4.13 (95% CI 3.67–4.65)	2.03 (95% CI 1.91–2.16)	8.52	89.3%
Female	0.23 (95% CI 0.18–0.28)	4.54 (95% CI 3.83–5.28)	2.13 (95% CI 1.96–2.30)	8.94	88.0%
Male	0.25 (95% CI 0.19–0.30)	3.66 (95% CI 3.10–4.34)	1.91 (95% CI 1.76–2.08)	8.02	90.8%

Abbreviations: MSE, mean squared error; RMSE, root mean squared error.

* Mean values of the fivefolds.

We then evaluated the performance separately in females and males: As expected, the *R* ^2^ dropped to, respectively, 0.29 (95% CI 0.25–0.33) and 0.28 (95% CI 0.24–0.32). These data suggest that gender contributes to approximately 35% of the explained variability in the handgrip values, whereas the other variables account for about 30%. Moreover, we observed a difference between MSE and RMSE: In females, the values were, respectively, 27.73 (95% CI 24.46–31.45) and 5.27 (95% CI 4.95–5.61), whereas in males 62.67 (95% CI 57.43–68.74) and 7.92 (95% CI 7.58–8.29). Consistent results were observed in the test set. Coherently, 95% model prediction intervals in the validation set were 18.00–30.00 in females and 28.00–48.60 in males. Consistent values were observed in the test set (18.30–29.90 in females and 28.00–49.10 in males).

The standard RF model yielded similar performance (Table S2a). In our sensitivity analysis, the addition of calf circumference to handgrip prediction did not result in model improvement (Table S3 and Figure S5).

**FIGURE 3:**
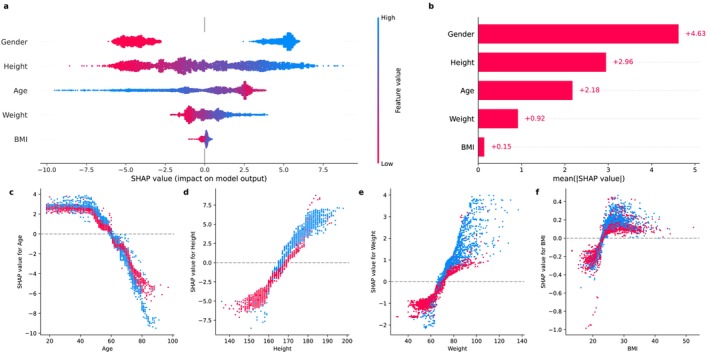
Variable contribution and interactions for handgrip on the validation set. (a) SHAP summary plot representing SHAP values (*x* axis) for each feature. Positive or negative SHAP values reflect whether the feature increases or decreases the model output, respectively. Each dot represents a subject, and the colour indicates the feature value. Male subjects are encoded as 1 and reported in blue. (b) Mean absolute SHAP values for each feature. (c, d) SHAP dependence plots exploring the relationship and interactions between features and their SHAP values, according to gender.

As expected, gender was the most important variable (absolute SHAP value: 4.63), followed by age and height (Figure 3a,b). Coherently, we observed an interaction between gender and age, as after 50 years, males had a steeper decrease in SHAP values compared to females. Interestingly, we observed a clear separation for BMI values above 25, whereas no clear difference was observed for lower values. A similar trend was observed for weight but not for height (Figure 3c–f).

We then assessed the association between RF residuals and PS (Table S4). In patients without PS, median value was 0.28 (IQR −2.98 to 3.89), compared to −7.39 (IQR −10.93 to −4.47) in patients with PS. This result was consistent according to gender: In subject with PS, median value was −6.41 (IQR −8.70 to −4.02) in females and −9.78 (IQR −14.50 to −5.22) in males. We then conducted an exploratory cutoff analysis to maximize the F1 score in the train set. In females, the optimal cut point was −6.08, and in males, it was −9.47, obtaining an F1 score in subject with PS of 0.46 and an F1 score in subjects without PS of 0.95 (Figure S6). We then evaluated these values in the validation set, obtaining an accuracy of 0.91, a F1 score of 0.95 in subjects without PS and of 0.46 in subjects with PS, with a ROC‐AUC of 0.87. Similar results were observed in the test set (Figure S7).

### 3.4 Chair Stand Test Model

**FIGURE 4:**
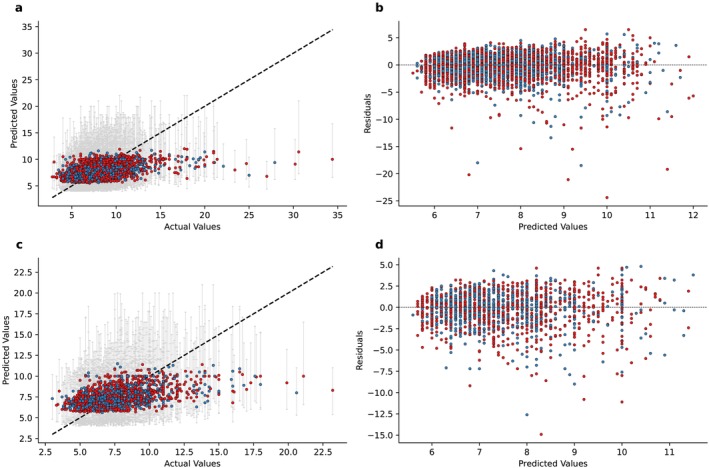
Model evaluation: chair stand test. (a) Comparison between predicted values (*y* axis) and actual values (*x* axis) in the validation set. Error bars indicate the 5th and 95th percentile. (b) Comparison between residuals (*y* axis) and predicted values (*x* axis) in the validation set. (c) Comparison between predicted values (*y* axis) and actual values (*x* axis) in the test set. Error bars indicate the 5th and 95th percentile. (d) Comparison between residuals (*y* axis) and predicted values (*x* axis) in the test set.

For CST prediction, the QRF model achieved a mean cross‐validated *R* ^2^ of 0.24 in the training set, with a MSE of 4.27 and a RMSE of 2.07 (Table 2b and Figure S8). In the validation set, the *R* ^2^ was 0.23 (95% CI 0.20–0.25), with a MSE of 4.68 (95% CI 4.12–5.32) and a RMSE of 2.16 (95% CI 2.03–2.31). Consistent results were observed in the test set: The *R* ^2^ was 0.24 (95% CI 0.20–0.28), with a MSE of 4.13 (95% CI 3.67–4.65) and a RMSE of 2.03 (95% CI 1.91–2.16). The MWIS scores were 8.70 (PC: 89.5%) in the validation set and 8.52 (PC: 89.3%) in the test set, with similar values in males and females. The actual versus predicted values plots and residual plots are shown in Figures 4a–d and S9.

We then evaluated the performance separately in women and men: the R^2^ remained stable to respectively 0.23 (95% CI 0.19–0.26) and 0.21 (95% CI 0.17–0.25). Moreover, we observed a difference between MSE and RMSE: In females, the values were, respectively, 5.22 (95% CI 4.40–6.25) and 2.28 (95% CI 2.10–2.50), whereas in males 4.02 (95% CI 3.41–4.67) and 2.00 (95% CI 1.85–2.16). Consistent results were observed in the test set. The 95% model prediction intervals were 5.84–10.40 in females and 6.02–10.00 in males in the validation set. Consistent results were observed in the test set: 5.80–10.14 for females and 6.06–10.00. The standard RF model yielded similar performance (Table S2b).

**FIGURE 5:**
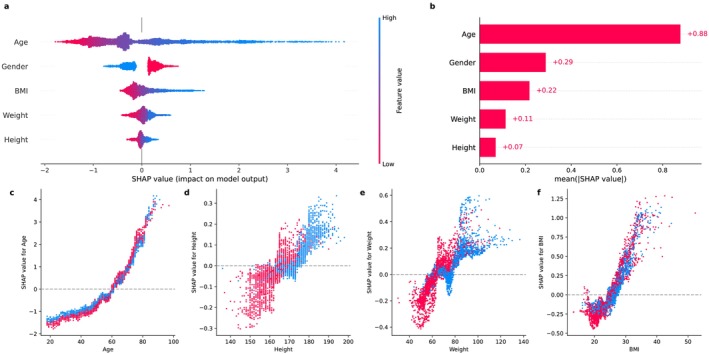
Variable contribution and interactions for chair stand test on the validation set. (a) SHAP summary plot representing SHAP values (*x* axis) for each feature. Positive or negative SHAP values reflect whether the feature increases or decreases the model output, respectively. Each dot represents a subject, and the colour indicates the feature value. Male subjects are encoded as 1 and reported in blue. (b) Mean absolute SHAP values for each feature. (c, d) SHAP dependence plots exploring the relationship and interactions between features and their SHAP values, according to gender.

As expected, age was the most contributing variable (absolute SHAP value: 0.88), followed by gender and BMI (Figure 5a,b). We observed an interaction between gender and age, as after 60 years, females had a steeper increase in SHAP values compared to males (Figure 5c–f).

We then assessed the association between RF residuals and PS (Table S5). In patients without PS, median value was −0.28 (IQR −1.25 to 0.87) compared to 0.60 (IQR −0.97 to 2.91) in patients with PS. This result was consistent according to gender: Median value in patients with PS was 0.85 (IQR −0.95 to 3.92) in females and 0.32 (IQR −1.02 to 2.41) in males (Figure S10). No further cutoff analysis was performed.

### 3.5 Models' Predictions and Integration

**FIGURE 6:**
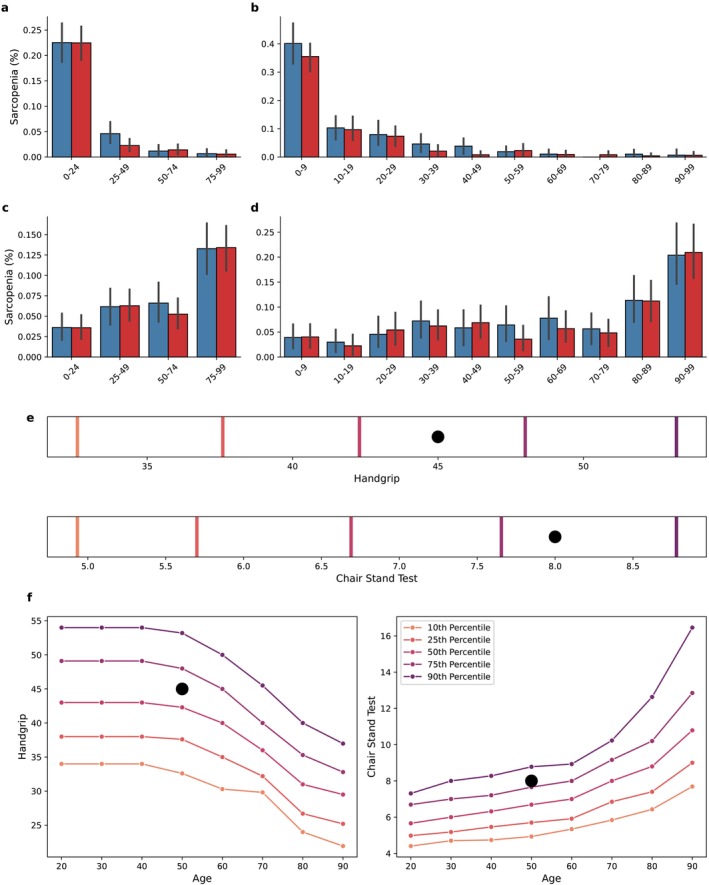
Quantile analysis and model predictions. (a, b) Percentage of patients with probable sarcopenia (according to EWGSOP criteria) according to predicted handgrip quartiles (a) and deciles (b) in the validation set. Error bars represent 95% confidence intervals. (c, d) Percentage of patients with probable sarcopenia (according to EWGSOP criteria) according to predicted chair stand test quartiles (c) and deciles (d) in the validation set. Error bars represent 95% confidence intervals. (e, f) Example of predictions for a single patient. Black dots represent the actual values. (e) The bars represent the 10th, 25th, 50th, 75th and 90th quartile for handgrip (upper) and chair stand test (lower). (f) The lines represent the 10th, 25th, 50th, 75th and 90th quartile keeping the patient characteristics fixed across different decades.

Model predictions were then interpreted in the context of EWGSOP2 cutoffs: We calculated the percentage of patients with PS according to EWGSOP2 criteria, stratified by the model‐derived predicted quantiles in the validation set. For handgrip, approximately 23% of individuals in the first predicted quartile met the criteria for PS, whereas this proportion dropped to less than 5% in the second predicted quartile. A similar decreasing trend was observed using deciles (Figure 6a,b). For CST, 13% of subjects in the 75th–99th percentile met the criteria of PS, whereas the prevalence was below 7% in the remaining quantiles. Using deciles, a notable increase was observed after the 80th percentile (Figure 6c,d).

Our proposed framework allows to visualize predicted outcomes alongside individualized reference quantiles, using subject‐specific characteristics as input. Figure 6e presents the output for a 50‐year‐old man, with weight 70 kg and height 175 cm. The actual values of handgrip and CST were 45 kg and 8 s, respectively. The black points represent the actual values and the vertical lines the corresponding quantiles. Figure 6f presents the corresponding quantiles for handgrip and CST test based on the individual's characteristics, showing the different quantiles for subject with the same baseline characteristics and different age.

## 4 Discussion

This study focused on developing predictive algorithms capable of estimating handgrip strength and CST test performance using basic demographic and anthropometric variables, such as age, sex, height, weight and BMI to support deployment in real‐world, community‐based scenarios. Currently, age and gender‐specific values falling below the 5th percentile for handgrip and above the 95th for the CST test are used to identify low muscle strength and PS, requiring additional evaluation [20]. Other cutoffs have also been proposed, such as the 25th percentile for handgrip and 75th percentile for CST [21]. This study extends our previous work [5], by shifting the paradigm from population level reference values to a subject‐specific approach, allowing the early identification of subjects of different ages at potential risk of sarcopenia, tailoring the timing of clinical or nutritional interventions, aiming to apply interventions earlier, when needed. This proactive approach may delay or even reverse the progression of sarcopenia, reducing the associated burden of frailty, falls, fractures and physical disability [22, 23]. To enhance applicability, a web‐based application is also under development to allow real‐time predictions for clinicians.

Compared to traditional regression models, tree‐based algorithms such as QRF offer specific advantages [7]. Due to the different statistical properties of the muscle strength measure, male and female are usually analysed separately, thus limiting the evaluation of possible interactions. In our analysis, the large amount of training data allowed the model to identify gender‐specific interactions and nonlinear relationships without prior specification: Inspecting the SHAP values of our model, we observed a differential decline in handgrip strength with age between males and females. Males demonstrated a higher variance of handgrip strength values and a steeper decline compared to females. The SHAP values for age started decreasing after 45 years both for males and females, crossing at approximately 60 years, followed by a steeper decrease in males. These two age cutoffs identified by our models align with established milestones in the aging process, as previously reported in studies exploring nonlinear patterns in molecular markers of aging [24]. Dysregulation around 44 and 60 years of age has been linked to significant transitions in physiological systems. Therefore, our findings further support the clinical relevance of an early screening for sarcopenia starting from middle age, with the aim of implementing appropriate strategies to prevent muscle strength loss in later years [25]. In this light, it is worth noting that currently used cutoffs are not applicable to young subjects and may therefore not be used for the early identification of young adults at risk for sarcopenia, a gap that our study tried to address. Moreover, our models indirectly validated the EWGSOP2 cutoffs for grip strength, with the lower bounds of the 95% prediction intervals closely aligning with established thresholds. This is relevant, because there was no information leakage to the model for the binary variable encoding PS. These models are also inherently robust to multicollinearity: Although BMI is derived from height and weight, at each split, the algorithm selects the most informative variable based on impurity reduction. SHAP analysis also confirmed that BMI, weight and height contributed nonredundantly, supporting their inclusion in the model. In the end, compared to standard approaches, nonparametric methods such as QRF are better suited to model the underlying variability observed in handgrip and CST performance, allowing data‐driven uncertainty estimation. Coherently, our model produced wider prediction intervals for handgrip in males than in females, reflecting the different variance. Clinically, this can inform the degree of confidence surrounding the predictions: Wider intervals may prompt more caution, additional testing or follow‐up assessments, particularly in borderline cases.

In our models, *R* ^2^ values for CST were generally modest compared to handgrip. Two main factors may contribute. Statistically, visual inspection of the residuals suggested that the CST model exhibited greater heteroscedasticity, suggesting more variability in prediction errors. Clinically, handgrip strength appears more sensitive to early declines in muscle function, whereas CST performance tends to remain within normal limits until later stages of decline. This could potentially make handgrip strength a better indicator across a wide range of functional statuses, whereas CST could have reduced sensitivity in detecting early changes. From a measurement perspective, CST performance is inherently more variable due to its dependence on factors beyond pure muscle strength, such as balance, joint mobility, motivation and environmental conditions. These nonanthropometric influences introduce additional noise that likely limits the model's explanatory power. It is important to recognize that quantile‐based models are not intended to provide single‐point predictions but rather to estimate conditional distributions, offering reference intervals that naturally incorporate prediction uncertainty [9]. To evaluate the reliability of these intervals, we used MWIS and PC, assessing how well the predicted intervals match the distribution of observed outcomes [18]. PC remained close to the nominal 90% threshold across datasets, supporting reliability of model's uncertainty estimates. In the end, although *R* ^2^ is not a direct measure of clinical utility, it remains one of the most interpretable metrics in regression tasks, quantifying the proportion of variance in the outcome explained by the model. A proper evaluation of clinical utility depends on how model outputs influence decisions and outcomes in real‐world practice, requiring an evaluation beyond the scope of the current study.

In addition to muscle strength, increasing attention has been directed towards muscle power [26], thanks to its earlier and faster decline during aging [27]. Recently, Alcazar et al. [28] proposed an equation to estimate lower extremity muscle power, based on the CST test, weight and height. Similar variables (age, sex and calf circumference) are currently used to estimate appendicular skeletal muscle, based on the equation developed by the COCONUT Study Group [29]. These data suggest that our choice of the included variables was sensitive and interestingly, in our sensitivity analysis, there was no improvement in the handgrip prediction by adding calf circumference.

To favour the application of these models in sarcopenia prescreening phase, we analysed the percentage of patients with PS according to predicted quantiles, and we tried to identify possible cutoffs both in males and females. Based on our results, we propose as possible cutoffs the 25th percentile for handgrip and 75th percentile for CST. A schematic workflow is presented in Figure S11. Further prospective evaluation is required to validate these specific thresholds for the prediction of confirmed sarcopenia. Interestingly, our cutoff analysis suggested that a simple difference between the actual and the predicted handgrip values could itself be a useful metrics to screen patients at higher risk. Recently, Yin and Zhao [30] proposed a 24‐variables gradient boosting classifier to predict sarcopenia based on the Asian Working Group for Sarcopenia 2019 framework. This model reached an AUC of 0.83 (95% CI 0.81–0.85), with an accuracy of 0.89 and an F1 score of 0.46. These results are in line with the ones achieved by our RF model using an absolute difference between the predicted and actual handgrip values greater than 6 kg for females and 9 kg for males. The application of artificial intelligence in sarcopenia research is rapidly evolving. A recent algorithm was developed in 2023, based on surface electromyography(sEMG), optimizing the support vector regression (SVR) model through a Sparrow Search Algorithm (SSA) [31]. The performance of the SSA‐SVR model was better than other regression prediction models, with an *R* ^2^ of 0.93 and a RMSE of 0.51. Although promising, this model relies on sEMG, an approach that requires specialized expertise and equipment. Subsequently, its applicability to community‐dwelling older adults is limited, and it may be less suitable for routine screening.

The integration of universally available variables within our algorithms enhances their adaptability across different settings, ensuring broader applicability. These predictions could facilitate routine screening procedures, and incorporating such predictive tools into electronic health records has the potential to streamline clinical workflows by automatically flagging individuals at elevated risk during routine healthcare visits, ensuring high‐risk patients are not overlooked. Additionally, functional measures such as handgrip strength and CST performance, when combined with the models' predictive outputs, may serve as surrogate biomarkers of the aging process, capturing functional decline.

Some limitations should be acknowledged. The predicted handgrip values and CST performance can only indicate the likelihood of PS, and a formal diagnosis requires confirmation, evaluating muscle quantity or quality, based on the EWGSOP2 criteria. Our cohort was predominantly composed of Caucasian participants, requiring validation of our findings in other ethnicities and geographic populations given the known differences in body composition, muscle strength and physical performance across different populations: External validation of our findings in multiethnic cohorts is essential to ensure a broader applicability and to refine population‐specific cutoffs. Quantiles were obtained from cross‐sectional data: If used longitudinally, quantiles cannot be interpreted as individual trajectories over time but should only be used to compare the predicted values for a subject with the same characteristics in different ages. A formal comparison with existing normative models, and an evaluation of whether the QRF‐based method improves the identification of individuals with confirmed sarcopenia, would require long‐term follow‐up data to prospectively assess the model's utility in long‐term risk stratification. Limitations also include the lack of information about specific conditions that could influence neuromuscular function, such as musculoskeletal and neurological disorders. The unconventional setting in which data was collected should be acknowledged. Although this recruitment strategy aligns with the intended use of the model as a prescreening tool to identify early functional decline in community‐dwelling adults, there is a potential for selection bias, as the population recruited may be relatively healthier and more functionally independent than the general adult population. Finally, the cutoff analysis for handgrip strength should be interpreted with caution, as it was exploratory in nature: The primary objective of our study was not to develop a binary classifier for PS but to predict muscle strength as a continuous outcome. The F1 score for PS was included to illustrate that even a model trained with few variables can yield reasonable performance in binary discrimination, despite not being specifically optimized for that task. Importantly, the observed performance is aligned with the intended role of the models as a prescreening tool rather than a confirmatory test. In this context, the high F1 score in individuals unlikely to have sarcopenia supports its use to reduce unnecessary testing in low‐risk populations.

These limitations aside, the developed models demonstrated the ability to predict subject‐specific quantiles for handgrip strength and the CST test using easily accessible variables such as age, sex, height, weight and BMI. By enabling personalized risk assessments, these models can facilitate the early identification of individuals at higher risk for sarcopenia, allowing timely interventions, which are likely to be more effective in preventing or delaying sarcopenia progression compared to interventions implemented during advanced stages of the condition.

## Ethics Statement

The study was conducted in accordance with the Declaration of Helsinki and approved by the Ethics Committee of the Università Cattolica del Sacro Cuore, Rome, Italy (Protocol No. A.1220/ce/2011). The authors of this manuscript certify that they comply with the ethical guidelines for authorship and publishing in the Journal of Cachexia, Sarcopenia and Muscle [32].

## Conflicts of Interest

The authors declare no conflicts of interest.

## Supporting information


**Table S1** Baseline variables in the train, validation and test set. Continuous values are represented as median [IQR]. Categorical variables as n (%). IQR: Interquartile range.
**Figure S1** Handgrip analysis of variance in the train set. **a.** Variance according to age bins. **b.** Percentage change according to age bins.
**Figure S2** Chair stand test analysis of variance in the train set. **a.** Variance according to age bins. **b.** Percentage change according to age bins.
**Figure S3** Model hyperparameter tuning summary for handgrip. **a.** R² values across consecutive optuna trials. **b.** Relationship between n_estimators and R² values, with point color representing max_depth and point size representing min_samples_leaf. **c.** Parallel coordinates plot for n_estimators, max_depth, min_samples_leaf according to R².
**Figure S4** Analysis of distributions and residuals for handgrip. **a, b, c:** validation set. **d, e, f:** test set. From left to right: distribution of predicted values, distribution of actual values, distribution of residuals.
**Table S2** Model performance of standard random forest (RF) regressor stratified by set and gender. *mean value of the 5‐folds. MSE: mean squared error, RMSE: root mean squared error. In validation set, 95% prediction intervals were 17.73‐30.89 in females and 28.22‐48.84 in males. In the test set, 95% prediction intervals were 18.30‐30.06 in females and 27.57‐50.47 in males.
**Table S3 Sensitivity analysis for handgrip adding calf circumference.** *mean value of the 5‐folds. MSE: mean squared error, RMSE: root mean squared error.
**Figure S5** Sensitivity analysis for handgrip adding calf circumference: variables contribution. **a.** SHAP summary plot representing SHAP values (x‐axis) for each feature. Positive or negative SHAP values reflect whether the feature increases or decreases the model output, respectively. Each dot represents a subject, and the color indicates the feature value. Male subjects are encoded as 1 and reported in blue. **b.** Mean absolute SHAP values for each feature.
**Table S4** Sarcopenia residuals comparison. The table reports the difference between the predicted and actual values for handgrip in the train set. Probable sarcopenia is defined by European Working Group on Sarcopenia in Older People 2 (EWGSOP2) criteria. Values reported are median and [IQR].
**Figure S6** Exploratory cut‐off analysis in the train set for handgrip. F1‐Score was used as target metric. In males the optimal cut‐off was ‐9.47 and in females ‐6.08. The F1‐Score in subject with PS was 0.46 and in subjects without PS 0.95. **a‐b** Distribution of the residuals. **c‐d** Thresholds plot evaluating performance metrics (precision, recall and F1 score) according to different threshold. Males are reported in blue on the left panels and females in red on the right panels.
**Figure S7** Confusion matrices (upper), ROC curve with 95% confidence interval (CI, middle) and PR‐AUC, using the cut‐off defined in the train set for handgrip. **a.** Train set. **b.** Validation set. **c.** Test set. In the validation set, accuracy was 0.91, F1 score 0.95 in subjects without PS and 0.46 in subjects with PS. The ROC‐AUC was 0.87 (95% CI: 0.85 ‐ 0.89) and the PR‐AUC: 0.42. Similarly, in the test set accuracy was 0.90, F1 score 0.95 in subjects without PS and 0.43 in subjects with PS. **The** ROC‐AUC: 0.88 (95% CI: 0.85 ‐ 0.91) **and the** PR‐AUC: 0.47. Confidence intervals were calculated using 2000 bootstrap replicates.
**Figure S8** Model hyperparameter tuning summary for chair stand test. **a.** R² values across consecutive optuna trials. **b.** Relationship between n_estimators and R² values, with point color representing max_depth and point size representing min_samples_leaf. **c.** Parallel coordinates plot for n_estimators, max_depth, min_samples_leaf according to R².
**Figure S9** Analysis of distributions and residuals for chair stand test. **a, b, c:** validation set. **d, e, f:** test set. From left to right: distribution of predicted values, distribution of actual values, distribution of residuals.
**Table S5** Sarcopenia residuals comparison. The table reports the difference between the predicted and actual values for chair stand test in the train set. Probable sarcopenia is defined by European Working Group on Sarcopenia in Older People 2 (EWGSOP2) criteria. Values reported are median and [IQR].
**Figure S10** Residual comparison in the train set for chair stand test. No cut‐off analysis was conducted. **a‐b** Distribution of the residuals. Males are reported in blue and females in red.
**Figure S11** Schematic representation of the Quantile Regression Forest (QRF) modelling workflow for functional assessment. Input variables include demographic and anthropometric predictors (age, gender, height, weight, BMI), with functional performance outcomes (handgrip and chair stand test). The QRF model generates individualized output including graphical and tabular quantile estimates, as well as patient‐specific age curves for interpretation of performance in a screening context.
**Table S6** TRIPOD‐AI checklist. 1. D=items relevant only to the development of a prediction model; E=items relating solely to the evaluation of a prediction model; D;E=items applicable to both the development and evaluation of a prediction model. 2. Separately for all model building approaches. 3. TRIPOD‐Cluster is a checklist of reporting recommendations for studies developing or validating models that explicitly account for clustering or explore heterogeneity in model performance (eg, at different hospitals or centres). Debray et al, BMJ 2023; 380: e071018 [DOI: 10.1136/bmj‐2022‐071018]. From: Collins GS, Moons KGM, Dhiman P, et al. BMJ 2024;385:e078378. doi: 10.1136/bmj‐2023‐078378.
